# Complete Genome Sequence of a Thermophilic Hydrogenotrophic Methanogen, *Methanothermobacter* sp. Strain CaT2

**DOI:** 10.1128/genomeA.00672-13

**Published:** 2013-08-29

**Authors:** Tomoyuki Kosaka, Hidehiro Toh, Atsushi Toyoda

**Affiliations:** Department of Biological Chemistry, Faculty of Agriculture, Yamaguchi University, Yamaguchi, Japana; PRESTO, Japan Science and Technology Agency (JST), Kawaguchi, Saitama, Japanb; Medical Institute of Bioregulation, Kyushu University, Higashi-ku, Fukuoka, Japanc; Center for Information Biology, National Institute of Genetics, Mishima, Shizuoka, Japand

## Abstract

We isolated a thermophilic hydrogenotrophic methanogen, *Methanothermobacter* sp. strain CaT2, which is able to aggregate and utilize formate. Here, we report the complete genome sequence of this organism.

## GENOME ANNOUNCEMENT

Hydrogenotrophic methanogens, which are obligate autotrophs, grow on H_2_ and CO_2_, some of which utilize formate, and are ubiquitous in a number of anaerobic environments (1). *Methanothermobacter* species are thermophilic hydrogenotrophic methanogens, occur largely in thermophilic methanogenic environments, and grow in a high temperature range, from 40 to 70°C, and in a pH range from 6.0 to 8.0 (1). Here, we report the complete genome sequence of a thermophilic and hydrogen- and formate-utilizing methanogen, *Methanothermobacter* sp. strain CaT2, isolated in our laboratory. CaT2 shows self-aggregation. CaT2 has been deposited in Deutsche Sammlung von Mikroorganismen und Zellkulturen GmbH (DSMZ) and NITE Biological Resource Center (NBRC) under the accession no. DSM 24414 and NBRC 107770, respectively.

The genome sequencing of CaT2 was performed by a 454 Life Sciences GS-FLX sequencer (Roche). Sequence reads were assembled with Newbler. A fosmid library (768 clones) was constructed. End sequencing of this fosmid library was performed by ABI 3730 capillary sequencers (Applied Biosystems). The 454 contig data and the fosmid end sequence data were assembled using the Phred-Phrap-Consed systems (2–4). Gap closing and resequencing of low-quality regions in the assembly data were performed by the nested deletion method (5), PCR, primer walking, use of shattered insert libraries (6), and direct sequencing of fosmid clones.

The genome sequence of CaT2 consists of a circular chromosome of 1,720,003 bp and an 11,015-bp plasmid. The plasmid, pCaT2, is nearly identical to the plasmid pFZ1 (11,014 bp, accession no. X68367) of *Methanothermobacter thermautotrophicus* strain Z-245 (7). The chromosome and the plasmid contain 1,749 and 11 predicted protein-coding genes, respectively. We compared the genome of CaT2 with those of the nonaggregating hydrogenotrophic methanogens *M. thermautotrophicus* ΔH (8) and *Methanothermobacter marburgensis* Marburg (9). Genome alignment showed a high level of sequence similarity and gene arrangements among the CaT2, ΔH, and Marburg strains. Sixty-seven genes in CaT2 were absent in both the ΔH and Marburg genomes, 35 (52%) of which encode hypothetical or conserved hypothetical proteins. The 67 genes include the *fdhABC* genes encoding formate dehydrogenase (MTCT_1438 and MTCT_1439) and formate transporter (MTCT_1440), which were not conserved in the ΔH and Marburg genomes; this is supported by a previous report that ΔH and Marburg are not able to grow on formate (1).

### Nucleotide sequence accession numbers. 

The sequence data for the CaT2 genome have been deposited in DDBJ/GenBank/EMBL under the accession no. AP011952 (chromosome) and AP011953 (plasmid).
